# Biosynthetic Pathway of Proanthocyanidins in Major Cash Crops

**DOI:** 10.3390/plants10091792

**Published:** 2021-08-28

**Authors:** Insu Lim, Jungmin Ha

**Affiliations:** Department of Plant Science, Gangneung-Wonju National University, Gangneung 25457, Korea; limis1014@gwnu.ac.kr

**Keywords:** proanthocyanidins, anthocyanins, parallel pathways, orthologous genes, cash crops

## Abstract

Proanthocyanidins (PAs) are a group of oligomers or polymers composed of monomeric flavanols. They offer many benefits for human fitness, such as antioxidant, anticancer, and anti-inflammatory activities. To date, three types of PA have been observed in nature: procyanidins, propelargonidins, and prodelphinidins. These are synthesized as some of the end-products of the flavonoid pathway by different consecutive enzymatic activities, from the same precursor—naringenin. Although the general biosynthetic pathways of PAs have been reported in a few model plant species, little is known about the species-specific pathways in major crops containing different types of PA. In the present study, we identified the species-specific pathways in 10 major crops, based on the presence/absence of flavanol-based intermediates in the metabolic pathway, and found 202 orthologous genes in the reference genomic database of each species, which may encode for key enzymes involved in the biosynthetic pathways of PAs. Parallel enzymatic reactions in the pathway are responsible for the ratio between PAs and anthocyanins, as well as among the three types of PAs. Our study suggests a promising strategy for molecular breeding, to regulate the content of PAs and anthocyanins and improve the nutritional quality of food sources globally.

## 1. Introduction

Proanthocyanidins (PAs), or condensed tannins, are oligomeric or polymeric end-products of flavonoid metabolism, starting from the central phenylpropanoid pathway [1]. PAs are brown-pigmented and present in the seed coats or seeds, fruits, bark, and leaves of a wide range of plant species, including important cash crops, such as apples, grapes, soybeans, common beans, cereals, and most berries [2]. These phytochemicals are brown-pigmented, increase plant resistance to herbivory, and protect plants from biotic and abiotic stresses, such as pathogens, insect attacks, and ultraviolet (UV)-B radiation [3,4].

In plants, PAs play important roles in resistance to several abiotic and biotic stresses [3,4]; several studies have reported that PAs increase tolerance to severe environmental conditions such as low temperature [5], drought [6], and UV-B radiation [7]. Furthermore, PAs impart astringency and bitterness to young leaves and fruits, deterring herbivory [8]. Accumulation of PAs also enhances tolerance to infection by biotrophic fungi [9,10] and other plant pathogens [7]. More PAs were accumulated after mechanical wounding and attack by herbivores [8].

PAs have attracted much attention because of their biological and therapeutic potential in humans. PAs provide unique flavors and acerbity to many foods and drinks, such as chocolate, fruit juice, tea, and wine [11]. Tea, which is the most commonly consumed beverage worldwide, is rich in PAs [12]. Cacao is a major food source with high PA content in the confectionery industry [13]. Pharmacological studies have shown beneficial effects of PAs in humans, such as antimicrobial, antidiabetic, antiaging, antioxidant, anticancer, and anti-inflammatory effects [14,15]. In addition to bioavailability, PAs have been reported to improve eyesight and neuroprotective functions, and promote flexibility in joints and blood circulation [16]. With these various benefits for human health, they have been considered to be important food-derived bioactive compounds in the pharmacological and cosmetic industries. Barks of common cinnamons are abundant with PAs, used as a fold medicine or supplement [17]. In soybeans, a cultivar containing high PA levels has been used as an ingredient for cosmetic products [18]. In grapes, because PAs are intensively accumulated in their seeds, seed oil products have been used as supplements for health promotion [19]. 

In the flavonoid biosynthetic pathway, PAs are the end-products of a branch of the anthocyanidin biosynthetic pathway. PAs and anthocyanins are derived from the same precursor—anthocyanidin—and share a common biosynthetic process for the conversion of phenylalanine to anthocyanidin [3]. PAs are a group of oligomers and polymers composed of flavan-3-ols—the most common subclass of flavonoids. Depending on the composition of the monomer precursor, the type of PA varies, including catechin, epicatechin, gallocatechin, epigallocatechin, afzelechin, and epiafzelechin, which are commonly found in the plant kingdom [2,20,21,22].

However, because of their beneficial properties, the accumulation of PAs has become a target of breeding and genetic engineering in a few model species; yet, the key genes involved in the pathway remain unclear in most cash crops [18]. In this study, species-specific biosynthetic pathways of PAs are reported based on metabolic intermediates, and orthologous genes of the key enzymes involved in each pathway are identified in 10 major cash crops. 

## 2. Biosynthetic Pathways of PAs and Anthocyanins

### 2.1. Chemical Structure of PAs

**Figure 1:**
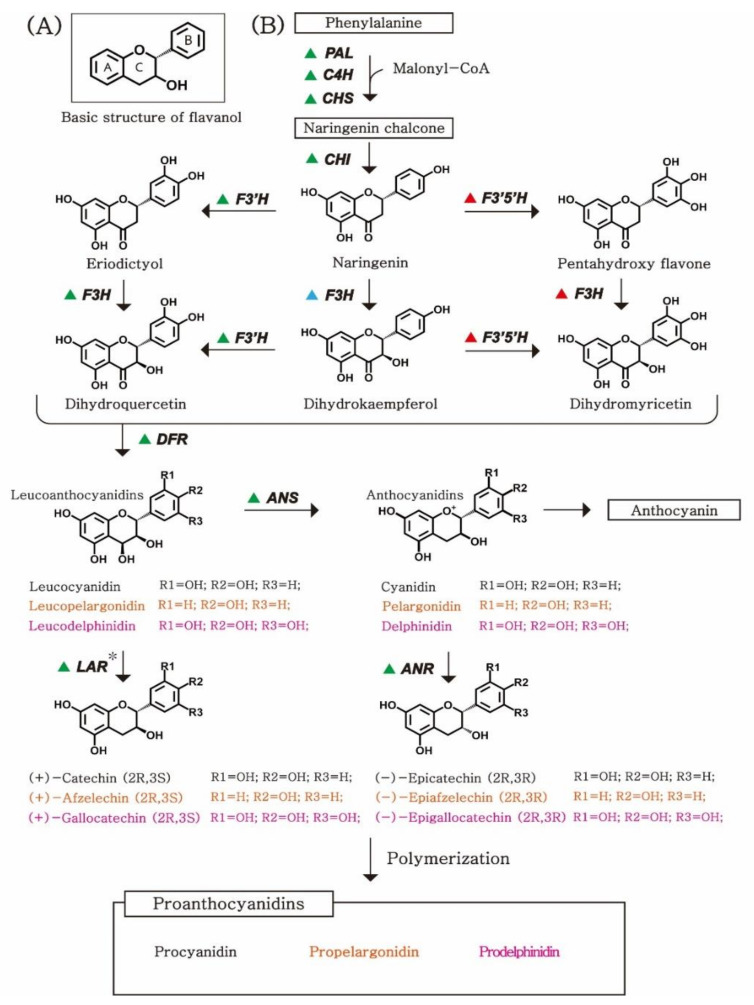
Biosynthetic pathway of PAs: (**A**) Basic chemical structure of flavan-3-ols. Flavan-3-ols have two benzene rings and one heterocyclic benzopyran ring. (**B**) A schematic diagram with chemical structures of flavanol compounds. Triangles indicate the species-specific enzymes involved in each biosynthetic pathway. The green triangle shows the enzymes that exist in all of our target species (Table 1); exceptionally, the LAR* gene, which catalyzes (2R, 3S)-flavanols such as catechin, was not identified in *A. thaliana*. A blue or red triangle indicates the enzyme that is present in grape, blueberry, and tea plant, or strawberry and almond, respectively. The names of the metabolites have been colored based on their hydroxyl pattern on the B-ring, and the black, orange, and pink designate 3′4′-dihydroxyl, 4′-hydroxyl, and 4′5′6′-trihydroxyl patterns, respectively. PAs: proanthocyanidins; ANR: anthocyanidin reductase; ANS: anthocyanidin synthase; LAR: leucoanthocyanidin reductase; F3′H: flavonoid 3′-hydroxylase; F3′5′H: flavonoid 3′,5′-hydroxylase; DFR: dihydroflavonol.

PAs are oligomers or polymers composed of flavan-3-ol units. The structure of PAs varies depending on the features of flavan-3-ols (as a starter or extension unit), the degree of polymerization [3,21], the position of the interflavan linkage between the monomeric units [4,21,23], and esterification of the 3-hydoxyl group [3,20,24,25]. Among these factors, the nature of flavan-3-ols (stereochemistry at the chiral center and hydroxyl patterns) is a key concept for understanding the structural variability of PAs. Flavan-3-ol units contain C6–C3–C6 basic flavonoid skeletons with two benzene rings, referred to as A and B rings, and one heterocyclic benzopyran ring, referred to as the C ring (Figure 1A) [26]. Flavan-3-ols have two chiral centers—the R and S configurations—on 2,3 of the C ring. Therefore, there are four diastereoisomers of flavan-3-ol with the same hydroxyl pattern: RR, RS, SR, and SS. The most common types of flavan-3-ol that serve as the building blocks of PAs include 2,3-*trans*-(+)-catechin and 2,3-*cis*-(−)-epicatechin (epimer) [27].

Another key factor that determines the type of flavan-3-ol is the hydroxyl pattern. At the early stage of the PA biosynthetic pathway, the formation of the 3′-hydroxyl and 3′5′-hydroxyl groups on the B-ring of flavan-3-ols is catalyzed by flavonoid 3′-hydroxylase (F3′H) and flavonoid 3′5′-hydroxylase (F3′5′H), respectively (Figure 1B). Depending on the hydroxyl pattern of flavan-3-ols, PAs can be categorized as procyanidins, propelargonidins, or prodelphinidins, which are homo-oligomeric PAs, with catechin and/or epicatechin as building units, and a 3′4′-dihydroxyl pattern. Propelargonidins and prodelphinidins are oligomers and polymers composed of extension units of afzelechin and epiafzelechin, respectively, with a 4′-hydroxyl pattern, and gallocatechin and epigallocatechin, respectively, with 4′5′6′-trihydroxyl groups [28,29]. Although, theoretically, there can be more types of PA depending on the differences in the hydroxyl pattern on the B-ring, except for the three types of PAs (procyanidins, propelargonidins, and prodelphinidins), no other types have been found in nature; therefore, these will not be discussed further here [30].

### 2.2. Biosynthetic Pathway of PAs

PAs are synthesized as one of the end-products of the flavonoid pathway, which shares the biosynthetic pathway with anthocyanins, from phenylalanine to leucoanthocyanidins (Figure 1B). According to the reference pathway of phenylpropanoid and flavonoid biosynthesis in the KEGG pathway database (www.genome.jp, accessed on 26 February 2021), the biosynthetic pathway begins with the conversion of phenylalanine to naringenin chalcone through cis-trans formation and cyclization by consecutive reactions mediated by phenylalanine ammonia-lyase, cinnamic acid 4-hydroxylase, and chalcone synthase (CHS). The cyclization of naringenin chalcone catalyzes the formation of naringenin by chalcone isomerase (CHI) [42,43,44,45].

In the second step, the type of flavan-3-ol (based on the B-ring on the hydroxyl pattern) is determined by two enzymes: F3′H and F3′5′H [46]. F3′H and F3′5′H can catalyze the conversion of naringenin to eriodictyol and pentahydroxy flavanone, respectively. Subsequently, naringenin and its two derivatives (eriodictyol and pentahydroxy flavanone) are catalyzed to produce dihydroflavanols by flavanone 3′-hydroxylase (F3H) [47]. DFR is a key enzyme that determines the type of flavan-3-ol by catalysis of three dihydroflavanols—2,3-dihydroquercetin, dihydrokaempferol, and 2,3-dihydromyricetin—into leucoanthocyanidins [48].

In the last steps of the biosynthetic pathway, the end-products are determined by the expressional competition and/or the presence/absence of ANS, ANR, and leucoanthocyanidin reductase (LAR) in each species [49,50,51]. Leucoanthocyanidins are synthesized into anthocyanidins by ANS, or into (2R, 3S)-flavan-3-ols—such as catechin, afzelechin, and gallocatechin—by LAR. Anthocyanidins (cyanidin, pelargonidin, and delphinidin) can be converted into epicatechin, epiafzelechin, and epigallocatechin, respectively, by ANR.

In the investigation of the biosynthetic pathways to produce different types of PAs, the constituents of flavan-3-ols were identified through a literature review to identify species-specific pathways in 10 major crops and the model plant species *Arabidopsis thaliana*. Catechin and epicatechin are commonly detected in apples, almonds, blueberries, cacao, common beans, grapes, strawberries, soybeans, tea plants, and peanuts [21,22,52,53,54,55,56]. In contrast, *A. thaliana* has been reported to have only epicatechin [50,57,58,59]. Afzelechin and epiafzelechin are mainly detected in almonds and strawberries [52,60]. Epigallocatechin and gallocatechin are found in grapevines, blueberries, and tea plants [53,54,61]. Other crops—including apples, cacao, common beans, soybeans, and peanuts—have been reported to contain only (epi)catechin or trace amounts of (epi)afzelechin and (epi)gallocatechin [21,22].

### 2.3. Regulatory Mechanisms of Flavonoids in Model Plants

Two stereoisomers—the flavan-3-ols (+)-catechin (2,3-*trans*) and (–)-epicatechin (2,3-*cis*)—are the building blocks of PAs. (–)-Epicatechin serves as a more common extender unit in the plant kingdom [1,3]. These play a critical role in seed dormancy, germination, and longevity [62].

The regulatory mechanisms underlying anthocyanin and PA biosynthesis have been intensively studied in *A. thaliana* The contents of anthocyanin and PAs have been reported to be responsive to plant hormones [63], temperature [64], UV irradiation [65], and drought [66]. Using *A. thaliana*, transparent testa (symbolized *tt*) mutants—TT2, TT8, and TTG1—have been reported to play key roles in regulating PA accumulation in seeds [67]. Flowering locus *C* and flowering locus *T* regulate PA biosynthesis to modulate seed dormancy [68]. In vegetative tissues, MBW (MYB-bHLH-WD repeat) complexes have been reported to regulate the anthocyanin biosynthetic pathway: MYB proteins including production of anthocyanin pigment 1 (PAP1)/MYB75, PAP2/MYB90, MYB113, and MYB114; and bHLH proteins including GLABRA3 (GL3), enhancer of GLABRA3 (EGL3), TT8, and WD-repeat protein TTG1 [67,69]. Leucoanthocyanidins—the common precursors of flavonoids in *A. thaliana*—are synthesized by CHS, CHI, F3H, F3′H, and DFR. Leucoanthocyanidin dioxygenase/anthocyanidin synthase (LDOX/ANS) and BANYULS/anthocyanidin reductase (BAN/ANR), TT19, TT12, and AHA10 are involved in the biosynthesis of PAs and anthocyanins from leucoanthocyanidins [70]. *DFR*, *LDOX*, *BAN*, *TT19*, *TT12*, and *AHA10* are directly targeted by the MBW complexes, and MYB11, MYB12, and MYB111 transcription factors regulate the transcription of *CHS*, *CHI*, and *F3H* [67,71].

*M. truncatula* and tobacco have also been well studied for bioengineering of PAs and anthocyanins [72]. When the *MtANR* gene was overexpressed, the levels of PAs were significantly increased, while the levels of anthocyanins were reduced in *M. truncatula* [72]. In *V. vinifera*, the *VvLAR1* gene was functionally characterized, and overexpression of *VvLAR1* in tobacco resulted in an increase in PA levels [73]. Similarly, the expression of the *CHS*, *CHI*, *F3H*, and *DFR* genes was reported to be upregulated by the *VvMYB5a* gene in tobacco [74].

Because the end-products of flavonoids are accumulated in the testa as pigments, and the levels of each flavonoid are significantly associated with seed coat color, studies on flavonoids have been intensively conducted in model legume species, where seed coat color varies and genomic information has been relatively well constructed. In soybeans, the color of seed coats ranges from yellow, green, buff, and brown to black, resulting from a combination of anthocyanin and PA compounds [75]. At least six loci—*I*, *T*, *Wp*, *W1*, *R*, and *O*—have been reported to be involved in this effect, and all of the genes corresponding to each locus have been characterized, including the *CHS, F3′H, F3H, F3′5′H, ANR, and R2R3 MYB* transcription factor genes [76,77,78,79,80,81]. Measurements of flavonoid compounds in various soybean germplasms have indicated that there are large variations in anthocyanin and PA levels, and that wild soybeans (*Glycine soja*), in general, have higher PA levels than cultivated soybeans (*G. max*) [82]. Black and red soybeans have been reported to possess catechin, epicatechin, and cyanidin 3-glucoside, whereas no PAs have been detected in yellow and buff soybeans. Brown soybeans have only PAs, but no anthocyanins [38,75,83,84]. IT109098—a *G. max* genotype with a brown seed coat—has been reported to have PA levels as high as those of *G. soja* [18]. It has been reported that the differences in the expression levels of genes encoding delphinidin 3-*O*-glucoside 2″-*O*-glucosyltransferases and ANR result in variations in the levels of anthocyanin and PA between the three genotypes with different seed coat colors: cultivated soybean (yellow), landrace (brown), and wild soybean (black).

### 2.4. Major Cash Crops with High PA Contents

In this study, a total of 10 high-PA-content cash crops with currently available genomic databases were selected: almond (*Prunus dulcis*), apple *(Malus domestica*), blueberry (*Vaccinium corymbosum*), cacao bean (*Theobroma cacao*), common bean (*Phaseolus vulgaris*), grape (*Vitis vinifera*), peanut (*Arachis hypogaea*), soybean (*Glycine max*), strawberry (*Fragaria* × *ananassa*), and tea tree (*Camellia sinensis*) (Table 1). These crops play a critical role in the global food market and cosmetic industry; according to statistical data from the Food and Agriculture Organization of the United Nations (www.fao.org, accessed on 5 February 2021), apples, grapes, and soybeans were ranked in the top 20 for commodity production in the USA in 2019. Almonds and blueberries were also produced at capacities of about 193.7 kt and 30.9 kt, respectively, in 2019 in the USA. Peanuts and strawberries are important commercial crops in several countries, including China, India, Indonesia, and the USA. In China, which has the largest market, the trade volumes of peanuts and strawberries were USD 471 million and 75 million, respectively, in 2019. Cacao beans are consumed worldwide as an ingredient in a variety of processed products—such as chocolate, cocoa powder, and cocoa butter—at a capacity of 3991 kt (in 2016, with the latest available data), as reported by Statista (www.statista.com/, accessed on 6 February 2021).

The PA content ranged from 145.0 to 3532.2 mg/100 g among the 10 species (Table 1). Grape seeds have been reported to have the highest level of PAs (3532.2 g/100 g), followed by cacao and common beans (1460.0 g/100 g and 1000.1 g/100 g, respectively) [2,14,34]. The levels of PAs detected in the 10 species can vary depending on the cultivars or measuring conditions used in each study, and the amount of actual absorption by humans can change depending on the manner of intake as food; for example, fresh grapes have much lower levels of PAs (0.05 g/100 g) than grape seeds (3532.2 g/100 g) [2]; in soybeans, seed coats contained significantly higher contents of PAs than the embryos [38]. In common beans, the levels of anthocyanin and PAs vary according to the color features of the bean coats, depending on the genotypes [34,85].

### 2.5. Identification of Orthologous Genes Involved in PA Biosynthesis in Major Cash Crops

Despite the nutritional importance of flavonoids, key enzymes involved in these pathways have not been identified in many major food crops with high flavonoid contents (Table 1). To characterize the PA biosynthetic pathways in the selected species, the orthologous genes encoding key enzymes involved in all pathways were searched in the latest reference genomic databases of each species (Table 1). N50 and genome coverage are two of the most important factors for evaluating the quality of genome assembly, where N50 is defined as the length of the contig, scaffold, super-scaffold, and pseudomolecule together being shorter than or equal to 50% of the total genome assembly length, and coverage is calculated from the percentage of the total assembly size over the reference genome. In this study, contig N50 varied widely, ranging from 79 kb (strawberry) to 1900 kb (common bean), and the highest, lowest, and mean genome coverage were 102% (blueberry and grape), 80% (common bean and cacao), and 94%, respectively (Table 1).

To identify orthologous genes involved in the PA pathway in the major crops, we collected a list of key enzymes (F3′5′H, F3′H, F3H, DFR, LAR, ANS, and ANR) based on the EC number from the KEGG database. Of the eight species, 92 genes were identified, including two wild relative species of strawberry and peanut (*Arachis duranensis*: 6, *A. thaliana*: 6, *Fragaria vesca*: 4, *G. max*: 20, *M. domestica*: 12, *P. vulgaris*: 12, *T. cacao*: 9, *V. vinifera*: 23) (Table 2). The protein sequences were downloaded from the NCBI GenBank database (www.ncbi.nlm.nih.gov, accessed on 4 March 2021) and cross-checked using the reference genomic database of each species (Table 2). We found that the sequences of three genes—XP_014632702.1 (*G. max*), XP_007138248 (*P. vulgaris*), and XP_017985307.1 (*T. cacao*)—were not available in the latest reference annotation data (Supplementary Table S1). In total, 83 genes were identified in the reference genome, and 14 paralogs, which had not been reported to be involved in the pathways in the KEGG database, were additionally detected in two species (*A. hypogea*: 5 and *F. ananassa*: 9; Table 2). Using these 97 genes, orthologous genes were identified in the eight crop species, and 105 genes were newly identified as candidate genes involved in the flavonoid biosynthetic pathway from five species (*A. hypogea*: 1, *C. sinensis*: 19, *F. ananassa*: 7, *P. dulcis*: 4, and *V. corymbosum*: 74) (Table 2, Supplementary Table S2). The annotation data currently available for the newly identified orthologous and paralogous genes agreed well with the EC numbers from the KEGG database. In *C. sinensis*, *P.* *dulcis*, and *V.* *corymbosum*, a total of 97 orthologs were first found in our study, and they have not been reported to be involved in the phenylpropanoid pathway according to the KEGG pathway.

### 2.6. Phylogenetic Analysis of Orthologs

**Figure 2:**
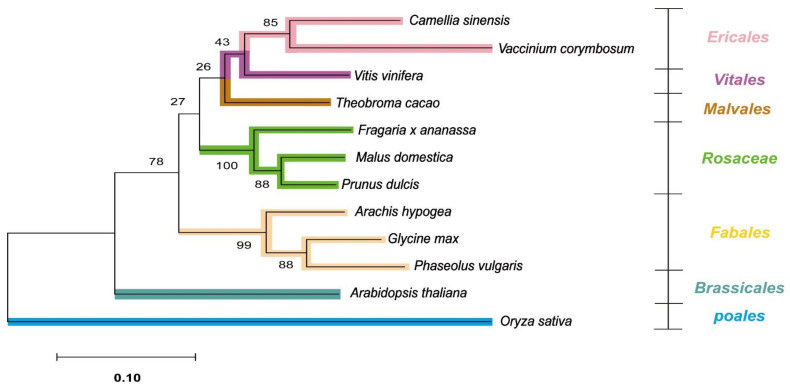
The phylogenetic tree was constructed using three genes: *ANR*, *ANS*, and *DFR*. The protein sequences of the three genes were obtained from the reference genomic data of the 12 species (including *Oryza sativa* as an outgroup). The protein sequences were concatenated and aligned using the MUSCLE algorithm, and the analysis was performed using the maximum likelihood method. The numbers between nodes are bootstrap values indicating the reliability of the genetic tree, and branches have been colored with their family indicated on the right-hand side of the figure. ANR: anthocyanidin reductase; ANS: anthocyanidin synthase; DFR: dihydroflavonol 4-reductase.

Using the three orthologous genes (*ANR*, *ANS*, and *DFR*), we conducted a phylogenetic analysis of the 12 species, including 10 cash crops, *A. thaliana*, and *Oryza sativa* as an outgroup. As expected, *O. sativa* was positioned outermost in the gene tree, and *A. thaliana* was also clearly distinguished from the other 10 major crops. In total, these 12 species could be categorized into 7 orders, and the phylogenetic tree agreed well with the taxonomy (Figure 2).

## 3. Regulatory Mechanisms for the Biosynthesis of PAs and Anthocyanins

PAs and anthocyanins are the two main classes of flavonoids, both of which have numerous beneficial effects on human health [15,16] and contribute to plant coloration, development, and responses to biotic and abiotic environments [86,87]. Over the last few decades, with advances in technologies to detect secondary metabolites, various types of flavanol-based metabolite have been identified in a large number of plant species [21,22]. Because of their diverse benefits, many studies have been performed to identify key enzymes involved in PAs in model plants [47,49,51,88,89]; however, the biosynthesis leading to the diversity of PAs has not been fully understood in most of the major crops cultivated worldwide.

### 3.1. Competitions between Parallel Pathways in the Flavonoid Pathway

In the flavonoid biosynthetic pathway, PAs and anthocyanins share a common biosynthetic pathway for the conversion of phenylalanine to anthocyanidin [3]. PAs and anthocyanins are derived from the same precursor—anthocyanidin—by the activity of ANR and anthocyanin 3′-*O*-beta-glucosyltransferase (3GT), respectively, [51,90]. PA and anthocyanin contents have been reported to be determined by the competition between these parallel pathways (Figure 1). Expression of *VvANR* increases the levels of PAs in grape fruit [73]. More PAs have been found to accumulate in the leaves of *MtANR*-overexpressing plants, as compared to those of the wild types of tobacco and *M. truncatula* [91]. In soybeans, the expression levels of ANR and 3GT have been shown to be associated with the levels of PAs and anthocyanins, respectively [18]. In blackberries, the expression levels of ANS and ANR have been found to correlate with the changing patterns of PA and anthocyanin levels [92]. We identified 71 orthologs of *LAR* (31), *ANS* (21), and *ANR* (19) (Table 3), thus curating a list of candidate genes that may play a critical role in regulating the levels of PAs or anthocyanins in major crop species.

The types of PA are also determined by another competition between parallel pathways. Different flavan-3-ol monomers are required as building blocks to polymerize the three types of PAs. Procyanidins, propelargonidins, and prodelphinidins are composed of (epi)catechin, (epi)afzelechin, and (epi)gallocatechin, respectively (Figure 1B) [93,94,95,96]. These monomeric building blocks are converted from the same precursor—naringenin—by consecutive enzymatic activities. The types of building blocks are determined by the location and number of hydroxyl groups on the B ring of flavan-3-ols. (Leuco)cyanidins and (leuco)delphinidins are catalyzed by eriodictyol and pentahydroxy flavone, respectively, which are hydroxylated by F3′H and F3′5′H from naringenin, respectively (Figure 1B). (Leuco)pelargonidins are catalyzed directly from naringenin, without hydroxylation. We presumed the enzymatic reactions occurring in each target crop based on the presence/absence of their metabolic intermediates, and detected the orthologous genes encoding key enzymes involved in the biosynthetic pathways of PAs.

### 3.2. Species-Specific Pathways for the Biosynthesis of Catechin and Epicatechin

Among PAs, procyanidin is the most abundant in the plant kingdom, and its monomeric building blocks—catechin and epicatechin—have also been reported as among the most common PAs [93,94]. These are also found in all of our target crops (Table 1), except for catechin, which has not been identified in *A. thaliana* to date [58,59,97]. This is consistent with our results, which showed that orthologous genes of *LAR* were not detected in *A. thaliana*, which catalyzed catechin from leucoanthocyanidins. In the present study, although both catechin and epicatechin were detected in *V. vinifera* and *P. dulcis* [21,22,52,53,73], orthologous genes of *ANR* were not detected in *V. vinifera*, while *F3H* and *F3′H* were not detected in *P. dulcis*. The complexity of their genome structure and/or the quality of the reference genome assemblies of *V. vinifera* and *P. dulcis* may impede the search for orthologous genes based on sequence similarity (Table 1). Alternatively, there might be species-specific pathways for the biosynthesis of catechin and epicatechin, which have not been reported even in model species.

Both procyanidins and prodelphinidins have been reported to accumulate in *C. sinensis*, *V. corymbosum*, and *V. vinifera* [2,53,61,98]. In contrast to Arabidopsis, the presence of *LAR* and the high activity of F3′5′H in *C. sinensis*, *Medicago truncatula, V. corymbosum*, and *V. vinifera* can promote the formation of dihydromyricetin, resulting in high PA content in leaf tissues [99]. In grapevines, the accumulation of procyanidins and prodelphinidins has been reported to depend on the presence of the two genes *VvF3*′*H* and *VvF3*′*5*′*H* [53,98]. Our analysis was also consistent with previous metabolic studies, which identified that most of the 49 orthologous genes of *F3*′*5*′*H* are present in the three species *C. sinensis* (5), *V. corymbosum* (37), and *V. vinifera* (7). In tobacco, the overexpression of *F3*′*5*′*H* increased delphinidin derivatives in transgenic plants, indicating that the existence of *F3*′*5*′*H* might be responsible for the high PA content in plants such as tea plants, blueberries, and grapes (Table 3).

Propelargonidins are the rarest group among PAs, because these are synthesized only when naringenin is not hydroxylated by either F3′H or F3′5′H (Figure 1). Among the 10 species, the orthologous genes of *F3*′*5*′*H* have not been detected in four species—*A. hypogea*, *F. ananassa*, *M. domestica*, and *P. dulcis*—where prodelphinidins have not been reported [21,22]. In *P. dulcis*, only propelargonidin and its monomer, afzelechin, have been detected, where both the orthologous genes of *F3*′*H* and *F3*′*5*′*H* have not been identified [21,22,52]. In *F. ananassa*, although orthologs of *F3*′*H* have been identified [21,22], both propelargonidins and procyanidins have been identified where the activity of the F3′H enzyme may be relatively low in the competition of parallel pathways.

In phylogenetic analysis, the three species with prodelphinidins—*C. sinensis*, *V. corymbosum*, and *V. vinifera*—were grouped closely, while the two species containing propelargonidins—*F. ananassa*, and *P. dulcis*—were also classified into the same clade as the Rosaceae family (Figure 2).

## 4. Conclusions

Although different types of PAs are present in many cash crops, PAs have been relatively underinvestigated compared to other flavonoids, such as anthocyanins. This study identified species-specific biosynthesis pathways for PAs and a list of responsive orthologs in the reference genomic data of each species. The competition between parallel pathways may represent a significant regulatory mechanism not only for PA content, but also for the types of PAs, depending on the species. Our results will play a role in molecular breeding, to improve the nutritional quality of dietary food sources. In addition to the food market, PAs have been receiving attention in the pharmacological and cosmetic industries, with therapeutic potential for humans. Furthermore, molecular engineering of the parallel pathways could be a prominent approach to regulate the PA and anthocyanin levels, depending on the desired purpose.

## 5. Materials and Methods

### 5.1. Sequence Analysis

The protein sequences of each enzyme corresponding to the enzyme commission number (EC number) in the Kyoto Encyclopedia of Genes and Genomes (KEGG) database were downloaded from the National Center for Biotechnology Information (NCBI) GenBank database (www.ncbi.nlm.nih.gov, accessed on 4 March 2021). Because these sequences had been uploaded by different research projects and, therefore, had different names even in the same species, these were blasted against the latest reference genomic databases of each species using BLASTP version 2.9.0, downloaded from the NCBI FTP server (ncbi.nlm.nih.gov/blast/executables/, accessed on 4 March 2021). A total of 92 genes from 8 species (*A. duranensis*, *A. thaliana*, *F. vesca G. max*, *M. domestica*, *P. vulgaris*, *T. cacao*, and *V. vinifera*) were used as queries to search for orthologous genes. Blast hits with percentage identity below 65% were removed (Table 2).

### 5.2. Phylogenetic Analysis

The protein sequences of three genes—anthocyanidin synthase (ANS), anthocyanidin reductase (ANR), and dihydroflavonol 4-reductase (DFR)—were used for phylogenetic analysis between 12 species, including *O. sativa* as an outgroup (*A. hypogea*, *A. thaliana*, *F. ananassa*, *G. max*, *C. sinensis*, *M. domestica*, *P. vulgaris*, *T. cacao*, *V. vinifera*, *P. dulcis*, and *V. corymbosum*). Orthologous genes were selected based on the *ANR* and *ANS* of cacao and *DFR* of peanuts, which are single genes. The sequences were concatenated and aligned using the MUSCLE algorithm, and conserved regions in the alignments were selected using Gblocks software (version 0.91) [100,101]. The analysis was then performed using alignments of a set of sequences, with the maximum likelihood method and MEGA X software (https://www.megasoftware.net/download_form, accessed on 9 April 2021). The phylogenetic tree was visualized using FigTree software version 1.4.4 (http://tree.bio.ed.ac.uk/software/figtree/, accessed on 14 April 2021) [102].

## Figures and Tables

**Table 1:** List of 10 major crops containing high levels of proanthocyanidins and their reference genomic information. Among major crops with high contents of PAs, the species with currently available genomic databases were selected for this study.

Species	Proanthocyanidins Content (mg/100 g)	Reference	Genome Database	Reference	Assembly Size (Mb)	Coverage (%)	Contig N50 (Kb)	Number of Genes Predicted
Almond (*Prunus dulcis*)	184	Prior & Gu, 2005 [14]	Prunus dulcis Lauranne Genome v1.0 (http://rosaceae.org/, accessed on 12 February 2021)	Alioto et al., 2020 [31]	227	95	103	27,969
Apple tree (*Malus domestica*)	162	Hellström et al., 2009 [2]	(iris.angers.inra.fr/gddh13/, accessed on 12 February 2021)	Daccord et al., 2017 [32]	643	100	620	42,140
Blueberry (*Vaccinium corymbosum*)	255	Prior & Gu, 2005 [14]	V_corymbosum v1.0 (http://gigadb.org/, accessed on 12 February 2021)	Colle et al., 2019 [33]	1680	102	15	32,140
Cacao bean (*Theobroma cacao*)	1460	Hellström et al., 2009 [2]	Cacao Matina1-6 Genome v2.1 (http://cacaogenomedb.org, accessed on 12 February 2021)	Publication in progress (http://cacaogenomedb.org)	346	80	1080	27,379
Common bean (*Phaseolus vulgaris*)	1000	Kan et al., 2016 [34]	Phaseolus vulgaris v2.1 (http://phytozome.jgi.doe.gov/, accessed on 12 February 2021)	Schmutz et al., 2014 [35]	600	80	1900	27,433
Grape (*Vitis vinifera*)	3532	Prior & Gu, 2005 [14]	Vitis vinifera v2.1 (http://phytozome.jgi.doe.gov/, accessed on 12 February 2021)	Jaillon et al., 2007 [36]	487	102	566	26,346
Peanut (*Arachis hypogaea*)	186	Hellström et al., 2009 [2]	(http://peanutgr.fafu.edu.cn/, accessed on 12 February 2021)	Zhuang et al., 2019 [37]	2538	94	1509	83,709
Soybean (*Glycine max*)	300	Lee et al., 2017 [38]	Glycine max Wm82.a4 (http://www.soybase.org, accessed on 12 February 2021)	Schmutz et al., 2010 [39]	1150	95	1492	46,430
Strawberry (*Fragaria × ananassa*)	145	Prior & Gu, 2005 [14]	(https://datadryad.org/, accessed on 12 February 2021)	Edger et al., 2019 [40]	813 *	99	79	108,087
Tea tree (*Camellia sinensis*)	189	Engelhardt et al., 2003 [12]	(http://tpia.teaplant.org/, accessed on 12 February 2021)	Xia et al., 2019 [41]	2890	95	67	53,512

* This assembly genome size (*Fragaria × ananassa*) is the haploid genome size.

**Table 2:** Numbers of genes involved in proanthocyanidins production in the 11 species. Based on sequence similarity, orthologous genes were searched using BLASTP.

Species	^a^ Number of Genes from KEGG Pathway	^b^ Number of Genes Confirmed from Reference Database	^c^ Number of Genes Newly Identified Using ^a^	Number of Orthologous Genes Newly Identified Using ^b+c^
*Arachis hypogea*	6 *	6	5	1
*Arabidopsis thaliana*	6	6	-	-
*Fragaria* × *ananassa*	4 *	4	9	7
*Glycine max*	20	19	-	-
*Camelia sinensis*	-	-	-	19
*Malus domestica*	12	12	-	-
*Phaseolus vulgaris*	12	11	-	-
*Theobroma cacao*	9	8	-	-
*Vitis vinifera*	23	17	-	-
*Prunus dulcis*	-	-	-	4
*Vaccinium corymbosum*	-	-	-	74
Total	92	83	14	105

* The genes involved in the biosynthetic pathways have been reported in wild relative species of strawberry (*Fragaria vesca*) and peanut (*Arachis duranensis*). ^a^ the number of genes identified from KEGG database, ^b^ the number ofgenes confirmed from reference database, ^c^ the number ofgenes newly identified from reference genome using ^a^.

**Table 3:** Numbers of the seven orthologs encoding key enzymes that synthesize PAs identified in the reference genome of each crop.

	*Arachis hypogea*	*Arabidopsis thaliana*	*Fragaria* × *ananassa*	*Glycine max*	*Malus domestica*	*Phaseolus vulgaris*	*Theobroma cacao*	*Vitis vinifera*	*Camelia sinensis*	*Prunus dulcis*	*Vaccinium corymbosum*
*F3*′*H*	2	1	3	5	2	1	1	2	1	0	4
*F3H*	2	1	4	4	2	1	1	2	2	0	8
*F3*′*5*′*H*	0	0	0	1	0	2	1	7	5	0	37
*DFR*	2	1	4	3	2	3	1	3	3	1	6
*LAR*	2	0	4	2	2	1	2	2	3	1	12
*ANS*	2	2	3	2	2	1	1	1	3	1	3
*ANR*	2	1	2	2	2	2	1	0	2	1	4
Total	12	6	20	19	12	11	8	17	19	4	74

## Data Availability

Data sharing not applicable.

## Supplementary Materials

The following are available online at https://www.mdpi.com/article/10.3390/plants10091792/s1. Table S1: List of genes involved in the biosynthetic pathways of PAs; Table S2: Identification of orthologous genes involved in PAs biosynthetic pathways.
