# Supplementary material for: Biosynthetic Pathway of Proanthocyanidins in Major Cash Crops
Source: Plants (Basel). 2021 Aug 28;10(9):1792. doi: 10.3390/plants10091792 (PMC8472070; doi:10.3390/plants10091792)
Supplement: Supplementary file 1 [file plants-10-01792-s001.zip › plants-1308862-supplementary.pdf]

Supplementary Table S1. List of genes involved in the biosynthetic pathways of PAs, as searched from KEGG database. The protein sequences were downloaded from NCBI GenBank database (www.ncbi.nlm.nih.gov), and cross-checked with the reference genomic database of each species using BLASTP.

Species	Query	Length of query	Subject	Length of subject	Enzymes	Percent identity	Alignments	Alignments/ subject length
AHP*	XP_015956243.1	340	AH03G35670.1	321	ANR	99	320	100
AHP*	XP_015956243.1	340	AH12G33420.1	340	ANR	98	340	100
AHP*	XP_015964353.1	353	AH05G08480.1	353	ANS	100	353	100
AHP*	XP_015964353.1	353	AH15G04650.1	353	ANS	100	353	100
AHP*	XP_020981632.1	312	AH16G40130.1	345	DFR	90	345	100
AHP*	XP_015945945.1	517	AH10G23440.1	517	F3`H	100	517	100
AHP*	XP_015945945.1	517	AH20G30480.1	517	F3`H	98	517	100
AHP*	XP_015952032.1	366	AH01G08350.1	366	F3H	100	366	100
AHP*	XP_015952032.1	366	AH11G00580.1	366	F3H	98	366	100
AHP*	XP_015967593.1	351	AH05G33160.1	351	LAR	99	351	100
AHP*	XP_015967593.1	351	AH15G19390.1	351	LAR	99	351	100
ATH	NP_176365.1	340	AT1G61720.1	340	ANR	100	340	100
ATH	NP_001154266.1	153	AT4G22870.2	153	ANS	100	153	100
ATH	NP_001031700.1	356	AT4G22880.1	356	ANS	100	356	100
ATH	NP_199094.1	382	AT5G42800.1	382	DFR	100	382	100
ATH	NP_190692.1	358	AT3G51240.1	358	F3H	100	358	100
ATH	NP_196416.1	513	AT5G07990.1	513	F3`H	100	513	100
FXA*	XP_004309662.1	341	maker-Fvb3-1-augustus-gene-304.62-mRNA-1	339	ANR	98	339	100
FXA*	XP_004309662.1	341	maker-Fvb3-2-augustus-gene-20.53-mRNA-1	339	ANR	98	339	100
FXA*	XP_004298720.1	383	maker-Fvb5-1-augustus-gene-7.57-mRNA-1	383	ANS	98	383	100
FXA*	XP_004298720.1	383	maker-Fvb5-2-augustus-gene-6.69-mRNA-1	397	ANS	95	393	99
FXA*	XP_004298720.1	383	maker-Fvb5-3-augustus-gene-266.54-mRNA-1	383	ANS	97	383	100
FXA*	XP_004287814.1	364	maker-Fvb1-1-augustus-gene-217.34-mRNA-1	364	F3H	99	364	100
FXA*	XP_004287814.1	364	maker-Fvb1-2-augustus-gene-78.37-mRNA-1	364	F3H	98	364	100
FXA*	XP_004287814.1	364	maker-Fvb1-3-augustus-gene-64.31-mRNA-1	364	F3H	98	364	100
FXA*	XP_004287814.1	364	maker-Fvb1-4-augustus-gene-56.40-mRNA-1	364	F3H	100	364	100

FXA*	XP_004297144.2	350	maker-Fvb4-1-augustus-gene-155.24-mRNA-1	350	LAR	97	350	100
FXA*	XP_004297144.2	350	maker-Fvb4-2-augustus-gene-44.51-mRNA-1	350	LAR	98	350	100
FXA*	XP_004297144.2	350	maker-Fvb4-3-augustus-gene-49.40-mRNA-1	350	LAR	100	350	100
FXA*	XP_004297144.2	350	maker-Fvb4-4-augustus-gene-44.47-mRNA-1	350	LAR	98	350	100
GMX	NP_001241913.2	337	glyma.Wm82.gnm4.ann1.Glyma.08G062000.1	337	ANR	100	337	100
GMX	NP_001243072.1	338	glyma.Wm82.gnm4.ann1.Glyma.08G062100.2	338	ANR	100	338	100
GMX	NP_001240884.1	352	glyma.Wm82.gnm4.ann1.Glyma.11G027700.1	352	ANS	100	352	100
GMX	NP_001239794.1	352	glyma.Wm82.gnm4.ann1.Glyma.01G214200.1	352	ANS	100	352	100
GMX	NP_001239696.1	339	glyma.Wm82.gnm4.ann1.Glyma.02G158700.1	339	DFR	100	339	100
GMX	NP_001238612.2	348	glyma.Wm82.gnm4.ann1.Glyma.14G072700.1	348	DFR	100	348	100
GMX	NP_001341095.1	354	glyma.Wm82.gnm4.ann1.Glyma.17G252200.3	354	DFR	100	354	100
GMX	XP_003520255.1	376	glyma.Wm82.gnm4.ann1.Glyma.02G048600.1	376	F3H	100	376	100
GMX	XP_003547939.1	372	glyma.Wm82.gnm4.ann1.Glyma.16G128700.1	372	F3H	100	372	100
GMX	XP_003517175.1	365	glyma.Wm82.gnm4.ann1.Glyma.01G166200.1	365	F3H	100	365	100
GMX	NP_001236797.1	375	glyma.Wm82.gnm4.ann1.Glyma.02G048400.3	375	F3H	100	375	100
GMX	XP_014632702.1			N/A				
GMX	NP_001236632.2	509	glyma.Wm82.gnm4.ann1.Glyma.13G072100.1	467	F3`5`H	99	463	99
GMX	XP_003525447.1	513	glyma.Wm82.gnm4.ann1.Glyma.05G021800.1	513	F3`H	100	513	100
GMX	XP_003525448.1	512	glyma.Wm82.gnm4.ann1.Glyma.05G021900.1	512	F3`H	100	512	100
GMX	XP_003525450.1	511	glyma.Wm82.gnm4.ann1.Glyma.05G022100.1	511	F3`H	100	511	100
GMX	XP_014624961.2	633	glyma.Wm82.gnm4.ann1.Glyma.17G077700.3	519	F3`H	99	515	99
GMX	NP_001237015.1	513	glyma.Wm82.gnm4.ann1.Glyma.06G202300.1	513	F3`H	100	513	100
GMX	NP_001352050.1	365	glyma.Wm82.gnm4.ann1.Glyma.20G185700.2	257	LAR	100	257	100
GMX	XP_003536310.1	363	glyma.Wm82.gnm4.ann1.Glyma.10G204800.2	363	LAR	100	363	100
MDM	NP_001280930.1	339	MD05G1335600	339	ANR	99	339	100
MDM	XP_028966264.1	339	MD10G1311100	339	ANR	100	339	100
MDM	NP_001280767.1	357	MD06G1071600	357	ANS	100	357	100
MDM	NP_001315877.1	357	MD03G1001100	410	ANS	99	357	87
MDM	NP_001280868.1	348	MD15G1024100	348	DFR	100	348	100
MDM	XP_008377381.2	347	MD08G1028600	347	DFR	100	347	100

MDM	NP_001280854.1	365	MD02G1132200	365	F3H	99	365	100
MDM	NP_001280883.1	365	MD15G1246200	365	F3H	100	365	100
MDM	XP_008374610.1	511	MD06G1201700	511	F3`H	100	511	100
MDM	XP_008393217.2	511	MD14G1210700	511	F3`H	100	511	100
MDM	NP_001280789.1	349	MD13G1046900	422	LAR	99	349	83
MDM	NP_001281043.1	354	MD16G1048500	354	LAR	100	354	100
PVU	XP_007159212.1	337	Phvul.002G218600.1.p	337	ANR	100	337	100
PVU	XP_007159213.1	337	Phvul.002G218700.1.p	337	ANR	100	337	100
PVU	XP_007158443.1	353	Phvul.002G152700.1.p	353	ANS	100	353	100
PVU	XP_007160734.1	354	Phvul.001G012700.1.p	354	DFR	100	354	100
PVU	XP_007160735.1	339	Phvul.001G012800.1.p	339	DFR	100	339	100
PVU	XP_007145529.1	329	Phvul.007G246200.1.p	329	DFR	100	329	100
PVU	XP_007156141.1	365	Phvul.003G261900.1.p	365	F3H	100	365	100
PVU	XP_007146174.1	528	Phvul.006G018800.3.p	385	F3`5`H	99	335	87
PVU	XP_007146137.1	505	Phvul.006G015400.2.p	452	F3`5`H	100	452	100
PVU	XP_007155243.1	364	Phvul.003G185500.3.p	368	F3`H	100	364	99
PVU	XP_007138248			N/A				
PVU	XP_007143793.1	366	Phvul.007G102100.1.p	348	LAR	94	366	105
TCC	XP_007025907.2	336	Thecc.06G207800.1.p	336	ANR	100	336	100
TCC	XP_007040068.2	354	Thecc.03G267700.1.p	354	ANS	99	354	100
TCC	XP_007017586.2	345	Thecc.08G030500.1.p	345	DFR	99	345	100
TCC	XP_017985307.1			N/A				
TCC	XP_007046698.1	364	Thecc.01G018700.1.p	364	F3H	100	364	100
TCC	XP_007015255.2	508	Thecc.09G247100.1.p	508	F3`5`H	99	508	100
TCC	XP_007037548.1	507	Thecc.03G100400.1.p	507	F3`H	100	507	100
TCC	XP_017971349.1	339	Thecc.02G361400.1.p	339	LAR	100	339	100
TCC	XP_017973773.1	359	Thecc.03G028300.1.p	359	LAR	100	359	100
VVI	NP_001268147.1	355	VIT_202s0025g04720.1	355	ANS	100	355	100
VVI	NP_001268144.1	337	VIT_218s0001g12800.1	375	DFR	100	337	90
VVI	XP_010664947.2	402	VIT_218s0001g12820.1	346	DFR	100	346	100

VVI	XP_019071960.1	339	VIT_218s0001g12810.1	410	DFR	100	337	82
VVI	NP_001268034.1	363	VIT_204s0023g03370.1	363	F3H	100	363	100
VVI	XP_002275563.1	358	VIT_218s0001g14310.1	358	F3H	100	358	100
VVI	NP_001268157.1	508	VIT_206s0009g02840.1	508	F3`5`H	99	508	100
VVI	XP_003632242.1	508	VIT_206s0009g03110.1	508	F3`5`H	99	508	100
VVI	XP_010651674.1	404	VIT_206s0009g02920.1	390	F3`5`H	99	305	78
VVI	XP_003632212.1	508	VIT_206s0009g02970.1	508	F3`5`H	100	508	100
VVI	XP_010651527.1	508	VIT_206s0009g02840.1	508	F3`5`H	100	508	100
VVI	XP_019075997.1	442	VIT_206s0009g02860.1	508	F3`5`H	97	422	83
VVI	XP_010651523.1	508	VIT_206s0009g02840.1	508	F3`5`H	97	508	100
VVI	XP_002280939.1	508	VIT_206s0009g02860.1	508	F3`5`H	97	508	100
VVI	XP_002271739.1	513	VIT_208s0007g05160.1	552	F3`5`H	100	513	93
VVI	XP_010651525.1	508	VIT_206s0009g02860.1	508	F3`5`H	100	508	100
VVI	XP_003632209.1	508	VIT_206s0009g03110.1	508	F3`5`H	100	508	100
VVI	XP_002280662.3	509	VIT_206s0009g03140.1	511	F3`5`H	100	509	100
VVI	NP_001268164.1	508	VIT_206s0009g02840.1	508	F3`5`H	97	508	100
VVI	NP_001267916.1	509	VIT_217s0000g07200.1	509	F3`H	99	509	100
VVI	XP_002284151.1	509	VIT_217s0000g07210.1	358	F3`H	100	358	100
VVI	NP_001268089.1	362	VIT_217s0000g04150.2	358	LAR	98	362	101
VVI	NP_001267887.1	346	VIT_201s0011g02960.1	346	LAR	100	346	100

AHP*, FXA*: The genes had been reported from the relative wild species of strawberry (*Fragaria x vesca*) and peanut (*Arachis duranensis*) in KEGG database.

PAs: proanthocyanidins, NCBI: National Center for Biotechnology Information, KEGG: Kyoto Encyclopedia of Genes and Genomes.

Supplementary Table S2. Identification of orthologous genes involved in PAs biosynthetic pathways A total of 108 genes were detected in the eight species. Protein sequences were downloaded from the NCBI GenBank database (www.ncbi.nlm.nih.gov), and orthologs were searched based on sequence identity from the reference genomic database of each species using BLASTP.

Species of query	Query	Length of query	Species of DB	Subject	Length of DB	Name of enzyme	Percent identity	Alignments	Alignments/ subject length
GMX	NP_001341095.1	354	AHP	AH16G40140.1	230	DFR	74	225	98
MDM	NP_001280930.1	339	CSS	TEA009266.1	349	ANR	79	333	95
TCC	XP_007025907.2	336	CSS	TEA022960.1	341	ANR	81	340	100
VVI	NP_001268147.1	355	CSS	TEA010322.1	307	ANS	85	307	100
TCC	XP_007040068.2	354	CSS	TEA015762.1	357	ANS	77	316	89
TCC	XP_007040068.2	354	CSS	TEA015769.1	575	ANS	82	346	60
VVI	XP_019071960.1	339	CSS	TEA023829.1	344	DFR	68	322	94
VVI	XP_019071960.1	339	CSS	TEA024758.1	343	DFR	69	334	97
MDM	NP_001280868.1	348	CSS	TEA032730.1	334	DFR	77	325	97
VVI	XP_010651525.1	508	CSS	TEA013315.1	486	F3\`5\`H	78	509	105
VVI	XP_002271739.1	513	CSS	TEA026294.1	506	F3\`5\`H	76	502	99
VVI	XP_002271739.1	513	CSS	TEA026296.1	506	F3\`5\`H	76	502	99
VVI	XP_003632209.1	508	CSS	TEA034021.1	470	F3\`5\`H	66	503	107
VVI	XP_010651525.1	508	CSS	TEA034051.1	507	F3\`5\`H	67	502	99
VVI	NP_001267916.1	509	CSS	TEA006847.1	518	F3\`H	80	516	100
VVI	XP_002275563.1	358	CSS	TEA023790.1	368	F3H	91	332	90
GMX	XP_003520255.1	376	CSS	TEA034016.1	320	F3H	77	123	38
VVI	NP_001268089.1	362	CSS	TEA021535.1	398	LAR	70	326	82
TCC	XP_017971349.1	339	CSS	TEA026458.1	326	LAR	71	316	97
TCC	XP_017971349.1	339	CSS	TEA027582.1	334	LAR	70	339	101
MDM	NP_001280868.1	348	FXA	maker-Fvb2-1-augustus-gene-255.45-mRNA-1	350	DFR	84	348	99
MDM	NP_001280868.1	348	FXA	maker-Fvb2-3-augustus-gene-33.39-mRNA-1	341	DFR	82	328	96
MDM	NP_001280868.1	348	FXA	maker-Fvb2-4-snap-gene-258.87-mRNA-1	355	DFR	80	328	92
MDM	NP_001280868.1	348	FXA	snap_masked-Fvb2-3-processed-gene-33.26-mRNA-1	368	DFR	81	366	99

MDM	XP_008393217.2	511	FXA	augustus_masked-Fvb5-2-processed-gene-78.0-mRNA-1	794	F3`H	85	501	63
MDM	XP_008393217.2	511	FXA	maker-Fvb5-1-augustus-gene-87.57-mRNA-1	512	F3`H	84	513	100
MDM	XP_008393217.2	511	FXA	snap_masked-Fvb5-3-processed-gene-202.30-mRNA-1	608	F3`H	76	323	53
MDM	NP_001280930.1	339	PDU	Prudu_010859_v1.0	624	ANR	90	339	54
MDM	NP_001280767.1	357	PDU	Prudu_014083_v1.0	381	ANS	92	357	94
MDM	XP_008377381.2	347	PDU	Prudu_003295_v1.0	344	DFR	86	344	100
TCC	XP_017973773.1	359	PDU	Prudu_015133_v1.0	337	LAR	69	330	98
VVI	XP_019075997.1	442	VCC	augustus_masked-VaccDscaff1-processed-gene-340.3-mRNA-1	813	F3`5`H	68	409	50
MDM	NP_001280930.1	339	VCC	maker-VaccDscaff133-augustus-gene-0.14-mRNA-1	333	ANR	82	332	100
MDM	NP_001280930.1	339	VCC	maker-VaccDscaff15-augustus-gene-178.21-mRNA-1	333	ANR	82	332	100
MDM	NP_001280930.1	339	VCC	maker-VaccDscaff19-augustus-gene-256.19-mRNA-1	333	ANR	81	332	100
MDM	NP_001280930.1	339	VCC	maker-VaccDscaff24-augustus-gene-201.24-mRNA-1	355	ANR	77	354	100
TCC	XP_007040068.2	354	VCC	maker-VaccDscaff22-augustus-gene-131.24-mRNA-1	362	ANS	82	355	98
TCC	XP_007040068.2	354	VCC	maker-VaccDscaff43-augustus-gene-236.29-mRNA-1	360	ANS	82	355	99
TCC	XP_007040068.2	354	VCC	maker-VaccDscaff46-augustus-gene-11.23-mRNA-1	360	ANS	82	355	99
VVI	NP_001268144.1	337	VCC	augustus_masked-VaccDscaff13-processed-gene-166.8-mRNA-1	345	DFR	79	330	96
VVI	XP_019071960.1	339	VCC	augustus_masked-VaccDscaff1613-processed-gene-0.0-mRNA-1	336	DFR	71	336	100
VVI	NP_001268144.1	337	VCC	augustus_masked-VaccDscaff32-processed-gene-199.0-mRNA-1	363	DFR	75	348	96
VVI	NP_001268144.1	337	VCC	augustus_masked-VaccDscaff42-processed-gene-96.9-mRNA-1	363	DFR	75	348	96
VVI	XP_019071960.1	339	VCC	maker-VaccDscaff12-snap-gene-66.28-mRNA-1	695	DFR	72	336	48
TCC	XP_007017586.2	345	VCC	maker-VaccDscaff30-augustus-gene-208.37-mRNA-1	243	DFR	80	221	91
VVI	XP_019075997.1	442	VCC	augustus_masked-VaccDscaff1-processed-gene-55.4-mRNA-1	211	F3`5`H	82	211	100
VVI	XP_002271739.1	513	VCC	augustus_masked-VaccDscaff21-processed-gene-	508	F3`5`H	72	512	101

				326.1-mRNA-1					
VVI	XP_019075997.1	442	VCC	augustus_masked-VaccDscaff26-processed-gene-34.8-mRNA-1	375	F3`5`H	82	211	56
VVI	NP_001268164.1	508	VCC	maker-VaccDscaff1-augustus-gene-56.30-mRNA-1	333	F3`5`H	77	333	100
VVI	XP_003632212.1	508	VCC	maker-VaccDscaff1-snap-gene-55.44-mRNA-1	1440	F3`5`H	77	512	36
TCC	XP_007015255.2	508	VCC	maker-VaccDscaff10-augustus-gene-348.25-mRNA-1	512	F3`5`H	76	510	100
VVI	XP_003632212.1	508	VCC	maker-VaccDscaff10-augustus-gene-349.23-mRNA-1	512	F3`5`H	76	512	100
VVI	XP_019075997.1	442	VCC	maker-VaccDscaff10-augustus-gene-94.16-mRNA-1	455	F3`5`H	68	420	92
VVI	NP_001268164.1	508	VCC	maker-VaccDscaff10-snap-gene-348.34-mRNA-1	1499	F3`5`H	76	512	34
TCC	XP_007015255.2	508	VCC	maker-VaccDscaff11-augustus-gene-60.22-mRNA-1	512	F3`5`H	72	512	100
VVI	XP_003632242.1	508	VCC	maker-VaccDscaff11-snap-gene-61.32-mRNA-1	461	F3`5`H	69	510	111
TCC	XP_007015255.2	508	VCC	maker-VaccDscaff15-augustus-gene-67.31-mRNA-1	512	F3`5`H	73	512	100
TCC	XP_007015255.2	508	VCC	maker-VaccDscaff15-augustus-gene-68.25-mRNA-1	516	F3`5`H	73	510	99
TCC	XP_007015255.2	508	VCC	maker-VaccDscaff15-augustus-gene-68.26-mRNA-1	516	F3`5`H	73	510	99
TCC	XP_007015255.2	508	VCC	maker-VaccDscaff19-augustus-gene-162.21-mRNA-1	512	F3`5`H	73	512	100
TCC	XP_007015255.2	508	VCC	maker-VaccDscaff19-augustus-gene-162.22-mRNA-1	510	F3`5`H	73	510	100
TCC	XP_007015255.2	508	VCC	maker-VaccDscaff24-augustus-gene-335.35-mRNA-1	512	F3`5`H	73	512	100
VVI	XP_003632209.1	508	VCC	maker-VaccDscaff24-augustus-gene-336.30-mRNA-1	510	F3`5`H	73	510	100
VVI	XP_002271739.1	513	VCC	maker-VaccDscaff26-augustus-gene-318.27-mRNA-1	508	F3`5`H	72	512	101
VVI	XP_010651674.1	404	VCC	maker-VaccDscaff26-snap-gene-34.47-mRNA-1	183	F3`5`H	71	191	104
VVI	XP_003632212.1	508	VCC	maker-VaccDscaff26-snap-gene-34.48-mRNA-1	1013	F3`5`H	77	512	51
VVI	XP_002271739.1	513	VCC	maker-VaccDscaff29-augustus-gene-305.28-mRNA-1	508	F3`5`H	72	512	101
VVI	XP_019075997.1	442	VCC	maker-VaccDscaff29-augustus-gene-34.34-mRNA-1	211	F3`5`H	81	211	100
TCC	XP_007015255.2	508	VCC	maker-VaccDscaff29-snap-gene-34.40-mRNA-1	632	F3`5`H	73	330	52
VVI	XP_003632212.1	508	VCC	maker-VaccDscaff5-augustus-gene-36.29-mRNA-1	601	F3`5`H	76	512	85
VVI	XP_019075997.1	442	VCC	maker-VaccDscaff5-augustus-gene-37.34-mRNA-1	215	F3`5`H	80	171	80
TCC	XP_007015255.2	508	VCC	maker-VaccDscaff5-augustus-gene-37.35-mRNA-1	512	F3`5`H	77	510	100
VVI	XP_003632212.1	508	VCC	maker-VaccDscaff5-augustus-gene-37.39-mRNA-1	512	F3`5`H	76	512	100
TCC	XP_007015255.2	508	VCC	maker-VaccDscaff5-augustus-gene-49.26-mRNA-1	511	F3`5`H	76	510	100
TCC	XP_007015255.2	508	VCC	maker-VaccDscaff5-snap-gene-37.45-mRNA-1	1232	F3`5`H	76	510	41

TCC	XP_007015255.2	508	VCC	maker-VaccDscaff8-augustus-gene-360.23-mRNA-1	512	F3`5`H	77	510	100
VVI	XP_003632212.1	508	VCC	maker-VaccDscaff8-augustus-gene-361.35-mRNA-1	512	F3`5`H	78	512	100
VVI	XP_003632212.1	508	VCC	maker-VaccDscaff8-snap-gene-360.28-mRNA-1	1021	F3`5`H	77	512	50
VVI	XP_003632212.1	508	VCC	maker-VaccDscaff8-snap-gene-361.42-mRNA-1	537	F3`5`H	76	512	95
VVI	XP_003632242.1	508	VCC	snap_masked-VaccDscaff5-processed-gene-318.17-mRNA-1	388	F3`5`H	69	411	106
VVI	XP_019075997.1	442	VCC	snap_masked-VaccDscaff8-processed-gene-361.12-mRNA-1	176	F3`5`H	82	176	100
VVI	NP_001267916.1	509	VCC	maker-VaccDscaff13-augustus-gene-238.27-mRNA-1	533	F3`H	81	514	96
VVI	NP_001267916.1	509	VCC	maker-VaccDscaff30-augustus-gene-161.25-mRNA-1	516	F3`H	80	514	100
VVI	NP_001267916.1	509	VCC	maker-VaccDscaff32-augustus-gene-159.26-mRNA-1	536	F3`H	80	519	97
VVI	NP_001267916.1	509	VCC	maker-VaccDscaff42-augustus-gene-154.36-mRNA-1	516	F3`H	80	514	100
VVI	NP_001268034.1	363	VCC	augustus_masked-VaccDscaff42-processed-gene-20.3-mRNA-1	359	F3H	85	363	101
VVI	NP_001268034.1	363	VCC	maker-VaccDscaff13-augustus-gene-41.36-mRNA-1	359	F3H	85	363	101
VVI	NP_001268034.1	363	VCC	maker-VaccDscaff16-augustus-gene-381.32-mRNA-1	366	F3H	90	346	95
VVI	NP_001268034.1	363	VCC	maker-VaccDscaff30-augustus-gene-297.38-mRNA-1	359	F3H	85	363	101
VVI	NP_001268034.1	363	VCC	maker-VaccDscaff31-augustus-gene-324.32-mRNA-1	366	F3H	87	346	95
VVI	NP_001268034.1	363	VCC	maker-VaccDscaff32-augustus-gene-323.35-mRNA-1	359	F3H	85	363	101
VVI	NP_001268034.1	363	VCC	snap_masked-VaccDscaff18-processed-gene-15.24-mRNA-1	373	F3H	85	353	95
VVI	NP_001268034.1	363	VCC	snap_masked-VaccDscaff7-processed-gene-48.23-mRNA-1	373	F3H	86	353	95
TCC	XP_017971349.1	339	VCC	maker-VaccDscaff14-augustus-gene-252.26-mRNA-1	351	LAR	72	333	95
TCC	XP_017971349.1	339	VCC	maker-VaccDscaff19-augustus-gene-121.20-mRNA-1	347	LAR	72	347	100
TCC	XP_017971349.1	339	VCC	maker-VaccDscaff2-augustus-gene-298.20-mRNA-1	351	LAR	72	333	95
TCC	XP_017971349.1	339	VCC	maker-VaccDscaff20-augustus-gene-119.25-mRNA-1	350	LAR	73	347	99
TCC	XP_017971349.1	339	VCC	maker-VaccDscaff25-augustus-gene-293.25-mRNA-1	351	LAR	72	333	95
TCC	XP_017971349.1	339	VCC	maker-VaccDscaff28-augustus-gene-227.23-mRNA-1	350	LAR	72	347	99
TCC	XP_017971349.1	339	VCC	maker-VaccDscaff28-augustus-gene-232.26-mRNA-1	350	LAR	73	347	99

TCC	XP_017971349.1	339	VCC	maker-VaccDscaff3-augustus-gene-115.16-mRNA-1	352	LAR	72	334	95
VVI	NP_001268089.1	362	VCC	maker-VaccDscaff35-augustus-gene-247.32-mRNA-1	341	LAR	67	346	101
VVI	NP_001268089.1	362	VCC	maker-VaccDscaff36-augustus-gene-103.16-mRNA-1	351	LAR	68	351	100
TCC	XP_017973773.1	359	VCC	maker-VaccDscaff4-augustus-gene-340.14-mRNA-1	203	LAR	74	182	90
VVI	NP_001268089.1	362	VCC	maker-VaccDscaff9-augustus-gene-315.13-mRNA-1	346	LAR	69	346	100

PAs: proanthocyanidins, KEGG: Kyoto Encyclopedia of Genes and Genomes.
